# When an external pilot is successful, should it be possible to transform it into an internal pilot by continuing recruitment into the full trial is ready? A case study of the cord pilot trial

**DOI:** 10.1186/1745-6215-16-S2-P15

**Published:** 2015-11-16

**Authors:** Lelia Duley, Angela Pushpa-Rajah, Lucy Bradshaw, Jon Dorling, Eleanor Mitchell

**Affiliations:** 1 Nottingham Clinical Trials Unit, University of Nottingham, Nottingham, UK; 2 Neonatal Unit, University of Nottingham, Nottingham, UK

## Background

The Cord Pilot Trial recruited for one year at 8 sites to assess feasibility of a definitive UK trial, comparing timing of cord clamping for very preterm births. This was a complex study, funded as part of an NIHR programme for applied research. This paper will present and discuss our experience of trying to transform an external pilot into an internal pilot.

## Methods

At one year, recruitment was above target and other pre-specified feasibility criteria had been met. The TSC advised that recruitment should continue whilst funding for the full trial was sought, to maintain momentum, avoid loss of equipoise, and maximise efficiency. This was strongly supported by sites, endorsed by the DMC, and agreed with funder and sponsor.

## Results

A pathway for submission to NIHR HTA was agreed, as the usual research led call would require pilot sites to continue for 18 months. In view of the timescales, full trial preparation continued in parallel with grant submission. The full stage application was rejected, and the pilot trial therefore closed. A closure plan was agreed with sponsor and TSC, to allow sites to either close immediately, or to randomise women who had given consent if they remained eligible. Recruitment and compliance were maintained during phase 2.

## Conclusions

When an external pilot trial is successful, transforming it into an internal pilot by continuing into the full trial may maximise efficiency and value for money, but is a challenge to achieve.

